# Intracranial hemorrhage in large vessel occlusion patients receiving endovascular thrombectomy with or without intravenous alteplase: a secondary analysis of the DIRECT-MT trial

**DOI:** 10.1136/jnis-2022-019021

**Published:** 2022-10-21

**Authors:** Xiaowei Hu, Yu Zhou, Johanna Ospel, Feirong Yao, Yizhi Liu, Hui Wang, Bo Li, Pinjing Hui, Pengfei Yang, Yongwei Zhang, Lei Zhang, Zifu Li, Pengfei Xing, Huaizhang Shi, Hongxing Han, Shouchun Wang, Qi Fang, Jianmin Liu, Direct-MT investigators

**Affiliations:** 1 Department of Neurology, The First Affiliated Hospital of Soochow University, Suzhou, China; 2 Neurovascular Center, Naval Medical University Changhai Hospital, Shanghai, China; 3 Department of Radiology, University Hospital Basel, Basel, Switzerland; 4 Department of Radiology, The First Affiliated Hospital of Soochow University, Suzhou, China; 5 Department of Interventional Radiology, The First Affiliated Hospital of Soochow University, Suzhou, China; 6 Department of Stroke Center, The First Affiliated Hospital of Soochow University, Suzhou, China; 7 Department of Neurosurgery, First Affiliated Hospital of Harbin Medical University, Harbin, China; 8 Department of Neurology, Linyi People's Hospital, Linyi, China; 9 Department of Neurology, The First Affiliated Hospital of Jilin University, Changchun, China

**Keywords:** Hemorrhage, Thrombectomy, Thrombolysis

## Abstract

**Background:**

Alteplase before thrombectomy for patients with large vessel occlusion stroke raises concerns regarding an increased risk of intracranial hemorrhage (ICH), but the details of this relationship are not well understood.

**Methods:**

This was a secondary analysis of the DIRECT-MT trial. ICH and its subtypes were independently reviewed and classified according to the Heidelberg Bleeding Classification. The effects of alteplase before thrombectomy on ICH and ICH subtypes occurrence were evaluated using logistic regression. Clinical and imaging characteristics that may modify these effects were exploratorily tested.

**Results:**

Among 591 patients, any ICH occurred in 254 (43.0%), including hemorrhagic infarction type 1 in 12 (2.1%), hemorrhagic infarction type 2 in 127 (21.7%), parenchymal hematoma type 1 in 34 (5.8%), parenchymal hematoma type 2 in 50 (8.6%), and other hemorrhage types (3a-3c) in 24 (4.1%). Similar ICH frequencies were observed with combined alteplase and thrombectomy versus thrombectomy only (134/292 (45.9%) vs 120/299 (40.1%); OR 1.27, 95% CI 0.91 to 1.75, P=0.16), but patients treated with alteplase had a higher parenchymal hematoma rate (51/287 (17.8%) vs 33/297 (11.1%); OR 1.75, 95% CI 1.08 to 2.85, P=0.024). In the adjusted model, difference in parenchymal hematoma occurrence between groups remained significant (adjusted OR 1.71, 95% CI 1.00 to 2.92, P=0.049). Patients with history of diabetes (P_interaction_=0.048), hypertension (P_interaction_=0.02), antiplatelet therapy (P_interaction_=0.02), anticoagulation therapy (P_interaction_=0.04), and statin administration (P_interaction_=0.02) harbored a higher ICH rate when they received combination therapy.

**Conclusions:**

Our data showed that in the DIRECT-MT trial, alteplase did not increase overall ICH for large vessel occlusion patients treated with thrombectomy, but it increased the parenchymal hematoma rate.

**What is already known on this topic:**

- Alteplase plus thrombectomy for large vessel occlusion stroke patients may be associated with an increased risk of intracranial hemorrhage (ICH).

**What this study adds**

- Alteplase did not increase overall ICH for large vessel occlusion patients treated with thrombectomy, but it increased the incidence of parenchymal hematoma.

**How this study might affect research, practice or policy**

- We explored which factors may modify the effect of intravenous alteplase on ICH and parenchymal hematoma, which could provide a starting point for future investigation.

## Introduction

Currently, combined treatment with intravenous alteplase and endovascular treatment (EVT) constitutes the standard of care for patients with large vessel occlusion stroke.^1 2^ However, the added value of intravenous alteplase in EVT candidates with large vessel occlusion stroke who present directly to an EVT-capable hospital has been recently questioned. One main concern for alteplase preceding EVT is a potentially increased risk of intracranial hemorrhage (ICH), one of the most feared complications in the treatment of acute ischemic stroke.

Several randomized trials compared the safety and efficacy of combined intravenous alteplase and EVT versus EVT alone. While the DIRECT-MT and DEVT trials showed non-inferiority of EVT alone compared with concurrent IV alteplase,^3 4^ the SKIP, DIRECT-SAFE, and SWIFT DIRECT trials failed to show non-inferiority of an EVT-only paradigm,^5–7^ and MR CLEAN-NO IV was unable to show either non-inferiority or superiority of EVT-only.^8^ However, a detailed analysis of hemorrhagic complications, and the association of alteplase with ICH subtypes, as well as factors that may modify the treatment effect of alteplase on ICH, have not yet been performed.

DIRECT-MT was the first randomized controlled trial evaluating EVT alone versus combined EVT and intravenous alteplase for large vessel occlusion in the anterior circulation in patients who presented directly to an EVT-capable center. The trial showed that EVT alone was non-inferior to combined EVT and intravenous alteplase (adjusted common odds ratio (acOR) 1.07, 95% CI 0.81 to 1.40).^4^ This secondary analysis aimed to describe ICH prevalence and subtypes with combined EVT and intravenous alteplase versus EVT alone, and to identify modifiable predictors of ICH in both groups that may allow for ICH prevention.

## Methods

The raw data underlying this analysis will be made available by the corresponding author on reasonable request after approval by the DIRECT-MT investigators and Human Genetic Resource Administration of China.

### Study design and patients

DIRECT-MT was a randomized, controlled, open-label trial, assessing non-inferiority of EVT alone versus combined intravenous alteplase and EVT (combination therapy) in patients from 41 academic tertiary care centers in China presenting directly to an EVT-capable center. Patient eligibility and the methods of the DIRECT-MT trial have been reported elsewhere.^4 9^

Patients meeting the inclusion criteria were randomly assigned in a 1:1 ratio to undergo EVT alone or EVT preceded by intravenous alteplase, at a dose of 0.9 mg per kilogram of body weight, administered within 4.5 hours after symptom onset.

All patients or their legal representatives provided written informed consent before randomization. The study protocol was approved by a central medical ethics committee and the research board of each participating center.

### Clinical data collection

Patient demographic and clinical characteristics were recorded at the time of enrolment. Clinical evaluation was repeated during the hospital stay, with National Institutes of Health Stroke Scale (NIHSS) measurements at 24 hours and 5–7 days or discharge. At 3 months, the patients’ clinical status was assessed using the modified Rankin Scale (mRS), a seven-point scale ranging from 0 (no symptoms at all) to 6 (death) via structured interviews that were performed in person or by telephone with the use of standardized forms by local trained physicians who were unaware of the trial-group assignments.

### Imaging analysis

All patients underwent both non-contrast computed tomography (NCCT) of the head and computed tomography angiography (CTA) of the head and neck vessels as standard care. Radiological follow-up with NCCT and CTA was performed at 24–72 hours, with an additional NCCT at 5–7 days. Reperfusion quality after EVT was assessed with the expanded Thrombolysis in Cerebral Infarction (eTICI) score on the last intracranial angiographic run during the procedure.^10^ All imaging was evaluated by an independent imaging core laboratory, by trained staff who were unaware of the treatment assignments and patient baseline characteristics.

ICH was classified according to the Heidelberg Bleeding Classification,^11^ which includes the following four categories: hemorrhagic infarction type 1 (HI-1), that is, scattered small petechiae, no mass effect; hemorrhagic infarction type 2 (HI-2), that is, confluent petechiae without mass effect; parenchymal hematoma type 1 (PH-1), that is, hematoma within the infarcted tissue occupying <30% without substantive mass effect; and parenchymal hematoma type 2 (PH-2), that is, ICH within and beyond infarcted brain tissue occupying 30% or more of the infarcted tissue with obvious mass effect. ICH outside the infarcted brain tissue or intracranial-extracerebral hemorrhage was divided into parenchymal hematoma remote from infarcted brain tissue, intraventricular hemorrhage, subarachnoid hemorrhage, and subdural hemorrhage. An independent adjudication committee that was blinded to treatment assignment adjudicated all hemorrhagic events and classified ICH into symptomatic (sICH) or asymptomatic (aICH) according to whether or not there was a deterioration in clinical status. sICH was diagnosed according to the Heidelberg Classification if the new observed ICH was associated with any of the following conditions: (1) NIHSS score increased >4 points than that immediately before worsening; (2) NIHSS score increased >2 points in one category; and (3) deterioration that led to intubation, hemicraniectomy, external ventricular drain placement, or any other major interventions.

### Statistical analysis

Analyses were performed based on the as-treated principle. Clinical and imaging baseline variables were reported for patients with versus without any ICH at follow-up NCCT, and were compared between the groups using *χ^2^* test or Fisher exact test for categorical variables and a *t-*test or Kruskal–Wallis test for continuous variables.

The association between ICH and ICH subtypes with patient outcome was estimated and described by unadjusted odds ratios derived from binary logistic regression models, whereby the patient group without ICH served as the reference category. The effects of treatment allocation (combination therapy vs EVT-only) on ICH and ICH subtype occurrence were evaluated using binary or ordinal logistics regression as appropriate. Adjusted analyses were also performed with significant predictors of ICH and ICH subtypes as covariates.Then, two-way multiplicative interaction terms were entered into the adjusted models to test for modification of alteplase effect on ICH and ICH subtypes by patient characteristics.

All analyses were performed with the use of SAS software, version 9.4 (SAS Institute). Adjustment for multiple comparisons was not performed in this secondary, exploratory analysis. All P values were two-sided, with a statistical significance level set at 0.05.

## Results

### Patient characteristics, ICH occurrence, and patient outcome

**Figure 1:**
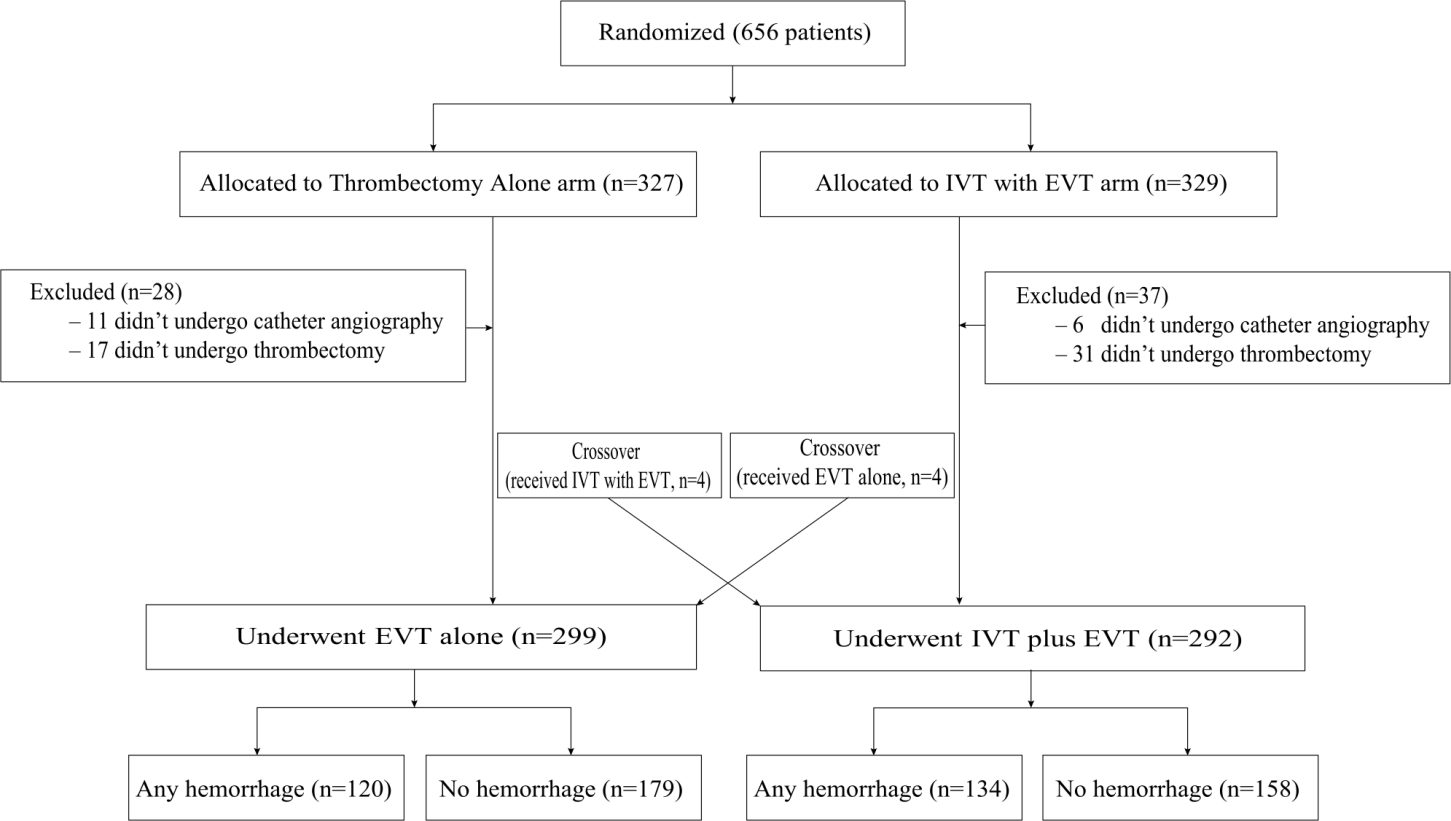
Flow diagram of included patients. EVT, endovascular treatment; IVT, intravenous thrombolysis.

Among 656 enrolled patients, 65 were excluded because of protocol violations or because they did not receive EVT (figure 1), leaving 591 patients for the final analysis. Three hundred and thirty-two (56.2%) patients were male, with a median age of 69 (interquartile range (IQR) 61–76) years. Two-hundred and ninety-two (49.4%) patients received combined intravenous alteplase and EVT, and 299 (50.6%) received EVT alone. Baseline characteristics of the overall patient sample and stratified by ICH (any ICH vs no ICH) are shown in online supplemental table S1. Baseline characteristics in the EVT-only group and combination therapy group were comparable (online supplemental table S2). Patients with ICH had higher baseline NIHSS scores, higher blood glucose levels, worse collateral circulation, lower clot burden scores, lower Alberta Stroke Program Early CT Score (ASPECTS), a higher proportion of internal carotid occlusion, and were treated with more thrombectomy maneuvers. They also had worse post-procedure eTICI scores and longer work-flow times (time from stroke onset to randomization, time from groin puncture to revascularization, time from onset to revascularization) in online supplemental table S1.

10.1136/jnis-2022-019021.supp1 Supplementary data

ICH occurred in 254/591 (43.0%) patients, of which 32/591 (5.4%) were symptomatic. ICH Heidelberg Bleeding Classification subtype information was lost in 7 patients; in the remaining 584 patients, HI-1 occurred in 12/584 (2.1%), HI-2 in 127/584 (21.7%), PH-1 in 34/584 (5.8%), PH-2 in 50/584 (8.6%), and other hemorrhage types in 24/584 (4.1%) (table 1). We were able to obtain the 90-day mRS in 590 patients (one patient with PH-2 was lost to follow-up). Compared with patients without ICH, ICH patients had lower rates of functional independence, that is, mRS 0–2 at 90 days (48/253 (19.0%) vs 166/337 (49.3%), OR 0.24 (0.17–0.35), P<0.001) and higher mortality (70/253 (27.7%) vs 38/337 (11.3%), OR 3.01 (1.95–4.65), P<0.001). Among the ICH patients, those with sICH and PH harbored the worst clinical outcomes. Functional independence rates in sICH patients were 1/31 (3.2%) (OR 0.034, 95% CI 0.005 to 0.26 compared with no ICH, P=0.001) and 13/83 (15.7%) in PH patients (OR 0.19, 95% CI 0.10–0.36 compared with no ICH, P=0.001), respectively (table 2).

**Table 1:** Hemorrhage distribution across the treatment groups

Heading	Overall (n (%))	Treatment		OR (95% CI)	P value
		Intravenous alteplase and EVT (n (%)) (n=292）	EVT-only (n (%)) (n=299)		
Any hemorrhage	254 (43.0)	134 (45.9)	120 (40.1)	1.27 (0.91 to 1.75)	0.16
Symptomatic ICH	32 (5.4)	20 (6.8)	12 (4.0)	1.90 (0.90 to 3.99)	0.095
Asymptomatic ICH	222 (37.6)	114 (39.0)	108 (36.1)	1.20 (0.85 to 1.68)	0.30
Hemorrhage type*					
HI-1	12 (2.1)	6/287 (2.1)	6/297 (2.0)	1.13 (0.36 to 3.58)	0.83
HI-2	127 (21.7)	61/287 (21.3)	66/297 (22.1)	1.05 (0.70 to 1.58)	0.83
HI total	139 (23.8)	67/287 (23.3)	72/297 (24.2)	1.05 (0.71 to 1.56)	0.79
PH-1	34 (5.8)	20/287 (7.0)	14/297 (4.7)	1.62 (0.79 to 3.31)	0.19
PH-2	50 (8.6)	31/287 (10.8)	19/297 (6.4)	1.85 (1.01 to 3.40)	0.048
PH total	84 (14.4)	51/287 (17.8)	33/297 (11.1)	1.75 (1.08 to 2.85)	0.024
Extra-parenchymal hemorrhage	24 (4.1)	11/287 (3.8)	13/297 (4.4)	0.96 (0.42 to 2.20)	0.92
No hemorrhage	337 (57.0)	158 (55.1)	179 0.3)	1	

*ICH Heidelberg Bleeding Classification subtype information was lost in 7 patients, 5 in intravenous alteplase and EVT group and 2 in EVT-only group.

aICH, asymptomatic intracranial hemorrhage; CI, confidence interval; EVT, endovascular thrombectomy; HI, hemorrhagic infarction; ICH, intracranial hemorrhage; OR, odds ratio; PH, parenchymal hematoma; sICH, symptomatic intracranial hemorrhage.

**Table 2:** Association between intracranial hemorrhage and patient outcome

Heading	Functional independence*			Mortality		
	N (%)	OR	P value	N (%)	OR	P value
No ICH (n=337)	166 (49.3)	1	NA	38 (11.3)	1	NA
ICH (n=253)	48 (19.0)	0.24 (0.17–0.35)	<0.001	70 (27.7)	3.01 (1.95–4.65)	<0.001
Symptomatic or not						
sICH (n=31)†	1 (3.2)	0.034 (0.005–0.26)	0.001	21 (67.7)	16.5 (7.2–37.7)	<0.001
aICH (n=222)	47 (21.2)	0.28 (0.19–0.41)	<0.001	49 (22.1)	2.23 (1.40–3.54)	0.001
ICH subtype‡						
HI (n=139)	28 (20.0)	0.25 (0.16–0.40)	<0.001	33 (23.6)	2.45 (1.46–4.11)	0.001
PH (n=83)	13 (15.7)	0.19 (0.10–0.36)	0.001	26 (31.3)	3.59 (2.02–6.37)	<0.001
Others (n=24)	8 (33.3)	0.52 (0.22–1.24)	0.14	6 (25.0)	2.62 (0.98–7.01)	0.055

*Functional independence denotes modified Rankin Scale (mRS) 0–2 at the 90-day follow-up.

†Patient follow-up was lost in 1 patient.

‡ICH Heidelberg Bleeding Classification subtype information was lost in 7 patients and patient follow-up was lost in 1 patient.

aICH, asymptomatic intracranial hemorrhage; HI, hemorrhagic infarction; ICH, intracranial hemorrhage; OR, odds ratio; PH, parenchymal hematoma; sICH, symptomatic intracranial hemorrhage.

### ICH and ICH types in patients with combination therapy versus EVT-only

As shown in table 1, rates of any ICH were similar between combination therapy versus EVT-only patients (134/292 (45.9%) vs 120/299 (40.1%), P=0.16). The same held true in patients with aICH or sICH. A nominally, although not significantly, higher rate of PH-1 (20/287 (7.0%) vs 14/297 (4.7%), P=0.19) and a significantly increased rate of PH-2 (31/287 (10.8%) vs 19/297 (6.4%), P=0.048) were observed in patients treated with combination therapy. The rate of any PH (PH-1 and PH-2) was also significantly higher with intravenous alteplase (51/287 (17.8%) vs 33/297 (11.1%), P=0.024), while the risk of any HI and extra-parenchymal hemorrhage did not differ between the groups.

Associations of other patient baseline and treatment variables with ICH and each ICH subtype in univariable analyses are shown in online supplemental tables S1, S3 and S4. Adjusted analysis by factors that contribute to ICH, PH, or sICH in univariate analysis yielded similar results. The difference in PH occurrence between groups remained significant after adjustment (adjusted odds ratio (aOR) 1.71, 95% CI 1.00 to 2.92, P=0.049) (table 1).

### Factors modifying alteplase effect on ICH and ICH subtypes

**Figure 2:**
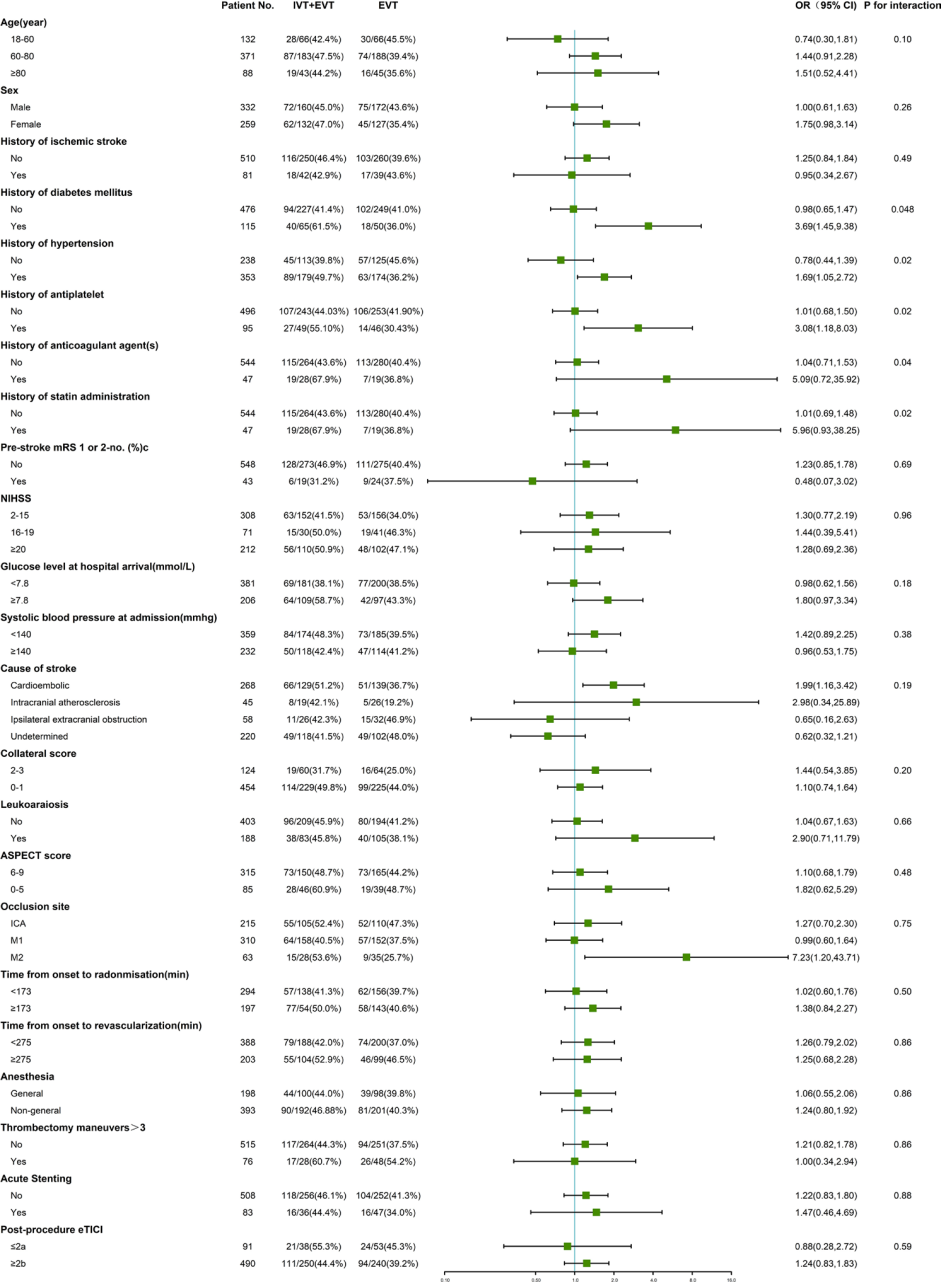
Forest plot of intracranial hemorrhage by subgroups, on as-treated population. NIHSS, the National Institutes of Health Stroke Scale, which ranges from 0 to 42 with higher scores indicating more severe neurologic deficits. Acute stenting indicated intracranial or extracranial stenting in DIRECT-MT. Analyses were adjusted by significant predictor factors for ICH in online supplemental file 1(baseline NIHSS score, baseline blood glucose level, worse collateral circulation, clot burden score, ASPECTS, occlusion site, thrombectomy maneuvers, post-procedure eTICI, and time from stroke onset to randomization, time from groin puncture to revascularization, time from onset to revascularization). EVT, endovascular treatment; ICH, intracranial hemorrhage; IVT, intravenous thrombolysis. ASPECTS, the Alberta Stroke Program Early CT Score, which is a measure of the extent of early cerebral ischemia. Scores range from 0 to 10, with higher scores indicating fewer early ischemic changes. eTICI, the extended Thrombolysis in Cerebral Infarction score, which ranges from 0 (no reperfusion) to 3 (complete reperfusion).

We performed exploratory analyses using adjusted regression models to investigate which factors may modify alteplase effects on ICH, sICH, and PH. History of diabetes, hypertension, antiplatelet therapy, anticoagulation therapy, and statin administration modified the treatment effect of intravenous alteplase on any ICH (figure 2). History of diabetes (aOR 3.69, 95% CI 1.45 to 9.38 vs aOR 0.98, 95% CI 0.65 to 1.47, P for interaction=0.048), history of hypertension (aOR 1.69, 95% CI 1.05 to 2.72 vs aOR 0.78, 95% CI 0.44 to 1.39, P for interaction=0.02), history of antiplatelet therapy (aOR 3.08, 95% CI 1.18 to 8.03 vs aOR 1.01, 95% CI 0.68 to 1.50, P for interaction=0.02), history of anticoagulation therapy (aOR 5.09, 95% CI 0.72 to 35.92 vs aOR 1.04, 95% CI 0.71 to 1.53, P for interaction=0.04), and history of statin administration(aOR 5.96, 95% CI 0.93 to 38.25 vs aOR 1.01, 95% CI 0.69 to 1.48, P for interaction=0.02) were associated with higher ICH rates in patients who received intravenous alteplase.

With regard to sICH, female patients experienced higher sICH rates when receiving combination therapy, but not male patients. (aOR 5.76, 95% CI 1.42 to 23.43 vs aOR 0.92, 95% CI 0.33 to 2.53, P for interaction=0.04). There was a borderline significant effect of anesthesia on sICH. In patients not treated under general anesthesia, the rate of sICH was higher with combination therapy (aOR 3.01, 95% CI 1.20 to 7.54), but not in those treated under general anesthesia (aOR 0.05, 95% CI 0.00 to 1.84, P for interaction=0.052) (online supplemental figure S1). We did not notice any factors that may modify the treatment effect on PH. Patients treated with acute stenting had higher rates of PH when treated with combination therapy (aOR 11.0, 95% CI 1.45 to 83.47), but the stenting-by-treatment interaction was not observed (P for interaction=0.13) (online supplemental figure S2).

## Discussion

This secondary analysis of the DIRECT-MT trial shows that hemorrhagic complications are common among patients receiving EVT (43% of the overall sample). Simultaneous administration of alteplase with EVT was associated with an increased risk of PH compared with EVT alone in both unadjusted and adjusted analyses (17.8% vs 11.0%; OR 1.75, 95% CI 1.08 to 2.85, P=0.024; aOR 1.71, 95% CI 1.00 to 2.92, P=0.049), but the risk of any ICH and sICH was not significantly increased.

Six randomized trials have compared simultaneous intravenous alteplase and EVT versus EVT-only in patients with large vessel occlusion stroke presenting directly to an EVT-capable hospital.^3–8^ However, they showed contradictory results on the occurrence of ICH following alteplase administration. In two trials enrolling Asian patients and with smaller sample sizes (SKIP and DEVT), a higher rate of any ICH was observed in patients treated with combined intravenous alteplase and EVT.^3 5^ In contrast, the DIRECT-MT results resemble those of the MR CLEAN-NO IV trial,^4 8^ which were very similar in terms of the trial design and sample size. Similarly, the recently published DIRECT-SAFE and SWIFT DIRECT trials also showed comparable ICH rates between the two treatment groups.^6 7^

We compared the baseline characteristics in the six published trials and attempted to explore the reasons for this discrepancy (online supplemental table S5). The nominally higher incidence of prior antiplatelet or anticoagulation therapy (20.7%/25% in DIRECT-MT vs 35%/34% in SKIP) may explain the higher ICH rate following combination therapy in SKIP, since history of antiplatelet or anticoagulation therapy was found to modify the treatment effect of intravenous alteplase on ICH in our study.^4 5^ However, the rate of prior antiplatelet or anticoagulation therapy was also high in MR CLEAN-NO IV (39.9% vs 40.6%).^8^ For the other trials, these data are not available at the present time. It is also unlikely that this discrepancy can be explained by a single variable. It is more likely caused by a combination of a multitude of factors such as ethnicity, sample size, prior antiplatelet or anticoagulation therapy, and time from onset to intravenous alteplase administration.

We further found that intravenous alteplase did not increase the incidence of any ICH in this study, but it did increase the risk of PH, and thus may increase not the frequency, but the severity of ICH. A similar finding was reported by Rozes *et al*.^12^ Furthermore, a pooled analysis of the ATLANTIS, ECASS, and NINDS trials showed a substantial increase in PH-2 rates in the alteplase group compared with the placebo group (5.9% vs 1.1%) in patients not treated with EVT.^13^ However, in the MR CLEAN-NO IV trial and SWIFT DIRECT trial, no such increase in hemorrhage severity was observed with combination therapy,^7 8^ which may again be explained by ethnic differences and other variables. Regarding sICH, we were not able to directly compare our data with the SKIP, SWIFT DIRECT, and DIRECT-SAFE trials because they defined sICH according to the NINDS, SIT-MOST, and the European Cooperative Acute Stroke Study criteria.^5–7^ In the other trials using the Heidelberg Bleeding Classification, conflicting results exist for the occurrence of sICH following intravenous alteplase in the setting of EVT. The DIRECT-MT, DEVT, and MR CLEAN-NO IV trials did not find any differences in sICH between the EVT-only and combined alteplase and EVT arms.^3 4 8^

The results of the current study are also somewhat inconsistent with another descriptive study by DIRECT-MT investigators, which analyzed the hemorrhagic distribution in the entire patient sample, and concluded that combination therapy did not increase the risk of hypertension, sICH, or PH, compared with EVT alone.^14^ The reason is that in the previous study, only 17 patients without catheter angiogram and 6 patients without large vessel occlusion were excluded, while in the present study, we performed an as-treated analysis. Furthermore, when evaluating the effect of alteplase on PH, the prior study used PH versus non-PH as categories, and found no association with alteplase and PH with a marginal P value (OR 1.57; 95% CI 0.98 to 2.50, P=0.06), while we compared PH to patients with no ICH at all.

In an exploratory analysis, we investigated which factors may modify the effect of intravenous alteplase on ICH, sICH, and PH as this could provide a starting point for future investigation. We found that patients with history of diabetes, hypertension, antiplatelet or anticoagulation therapy, and statin administration may experience higher risk of any ICH with intravenous alteplase. In other words, for these patients, EVT alone may be the preferred strategy. However, these analyses are exploratory, and the results should be interpreted with caution, especially in the analyses of sICH and PH, due to the rather low sample size and low prevalence of sICH and PH. For example, we observed higher sICH for female patients after combination treatment, which is inconsistent with previous studies.^15 16^Additional studies are needed to further investigate the associations that were observed in the exploratory subgroup analyses. Particularly in the case of borderline significant results like in the anesthesia subgroups, confirmatory studies in large datasets such as Highly Effective Reperfusion Evaluated in Multiple Endovascular Stroke Trials would be desirable to further consolidate the evidence from our study.^17^

With regard to patients who received intracranial stenting, the routine antiplatelet treatment protocol for intracranial stenting was periprocedural tirofiban followed by dual oral antiplatelet therapy (usually aspirin and a PY12 inhibitor) in the postprocedural period. However, the specific antiplatelet regimen was not recorded in the DIRECT-MT trial database and, as such, we could not directly include post-stenting antiplatelet therapy as a variable in the models to test whether the antiplatelet regimen, rather than the stenting itself, is the reason for the increased PH rates in patients treated with intracranial stenting.

Our study had some limitations. First, we conducted the analysis on the as-treated sample and included only those patients who received EVT, that is, some patients were excluded. Second, this was an exploratory analysis without adjustment for multiple testing, and the analysis may not have been sufficiently powered to detect factors that may affect ICH prevalence, patient outcomes, and modifiers of alteplase treatment effect. This is a potential reason for the borderline P value for the PH differences between groups in the adjusted analysis. Third, our study exclusively included Chinese patients who met the inclusion criteria of the DIRECT-MT trial, and further studies are needed to confirm the result in other ethnicities. Lastly, one should note that DIRECT-MT used the Heidelberg Bleeding Classification to classify bleeding events, and it is unclear whether the results would remain the same for ICH subtypes if the NINDS and SIT-MOST criteria for sICH were used, which also makes direct comparison with publications using the latter two classifications difficult.^18^

In conclusion, in this secondary analysis of a Chinese trial (DIRECT-MT), we found that in large vessel occlusion patients treated with EVT, intravenous alteplase in addition to EVT did not increase overall ICH, but it did increase the incidence of parenchymal hematoma.

10.1136/jnis-2022-019021.supp2 Supplementary data

## Data Availability

All data relevant to the study are included in the article or uploaded as supplementary information.
