# Supplementary material for: Intracranial hemorrhage in large vessel occlusion patients receiving endovascular thrombectomy with or without intravenous alteplase: a secondary analysis of the DIRECT-MT trial
Source: J Neurointerv Surg. 2022 Oct 21;15(10):977–82. doi: 10.1136/jnis-2022-019021 (PMC10511977; doi:10.1136/jnis-2022-019021)
Supplement: Supplementary data [file jnis-2022-019021supp001.pdf]

BMJ Publishing Group Limited (BMJ) disclaims all liability and responsibility arising from any reliance placed on this supplemental material which has been supplied by the author(s)

The Supplements list：

Table S1. Baseline Characteristics of the 591 included Patients by ICH or not.

Table S2. Baseline Characteristics of the 591 included Patients by treatment.

Table S3. Baseline Characteristics of the 591 included Patients by non-ICH versus aICH and sICH.

Table S4. Baseline Characteristics of the 591 included Patients by non-ICH versus HI, PH, and other ICH.

Table S5. Baseline characteristics of the patients of six randomized trials.

Figure S1. Forest plot of symptomatic intracranial hemorrhage by subgroups, on as-treated population.

Figure S2. Forest plot of parenchymal hematoma by subgroups, on as-treated population.

Hu X, *et al. J NeuroIntervent Surg* 2023; 15:977–983. doi: 10.1136/jnis-2022-019021

Table S1. Baseline Characteristics of the 591 included Patients by ICH or not[a].

Variable	All	ICH		P value
		Yes (n=254)	No (n=337)	
Age-yr, median (IQR)	69 (61-76)	69 (61-76)	70 (61-76)	0.54
Male sex-no. (%)	332 (56.2)	147 (57.9)	185 (54.9)	0.47
NIHSS-median (IQR)[b]	17 (13-22)	18 (14-23)	16 (13-21)	<0.001
History of ischemic stroke-no. (%)	81 (13.7)	35 (13.8)	46 (13.6)	0.96
History of atrial fibrillation-no. (%)	279 (47.2)	121 (47.6)	158 (46.9)	0.86
History of diabetes mellitus-no. (%)	115 (19.5)	58 (22.8)	57 (16.9)	0.072
History of hypertension-no. (%)	353 (59.7)	152 (59.8)	201 (59.6)	0.96
History of antiplatelet agent (s) -no. (%)	95 (16.1)	41(16.1)	54 (16.02)	0.97
History of anticoagulant agent (s) -no. (%)	47(8.0)	26 (10.2)	21 (6.2)	0.075
History of statin administration	47(8.0)	26 (10.2)	21 (6.2)	0.075
Pre-stroke modified Rankin scale, score 1 or 2-no. (%)[c]	43 (7.3)	15 (5.9)	28 (8.3)	0.27
Median glucose level at hospital arrival (IQR) —mmol/L	7.0 (5.9-8.8)	7.4 (6.2-9.7)	6.8 (5.8-8.1)	<0.001
Systolic blood pressure at admission -mmHg, median (IQR)	145 (130-163)	144 (131-161)	146 (130-163)	0.99
Cause of stroke -no. (%)[d]				
Cardioembolic	268 (45.3)	117 (46.1)	151 (44.8)	0.26
Intracranial atherosclerosis	45 (7.6)	13 (5.1)	32 (9.5)	
Ipsilateral extracranial obstruction	58 (9.8)	26 (10.2)	32 (9.5)	
Undetermined	220 (37.2)	98 (38.6)	122 (36.2)	
Collateral score-no. (%)[e]				
0-1	454 (76.8)	213/ (85.9)	241/330 (73.0)	<0.001
2-3	124 (21.0)	35 (14.1)	89/330 (27.0)	
Leukoaraiosis	188 (31.8)	78 (30.7)	110 (32.6)	0.62

Clot burden score		4 (2-5)	4 (2-5)	4 (3-5)	<0.001
ASPECTS-median (IQR)[f]		9 (7-10)	8 (6-9)	9 (7-10)	<0.001
Occlusion site[g]					
	ICA	215/588 (36.4)	107/252 (42.5)	108/336 (32.1)	0.036
	M1	310/588 (52.5)	121/252 (48.0)	189/336 (56.3)	
	M2	63/588 (10.7)	24/252 (9.5)	39/336 (11.6)	
Workflow- min, median (IQR)					
	Time from stroke onset to randomization	173 (127-211)	176 (132-221)	170 (124-203)	0.015
	Time from randomization to groin puncture	33 (21-47)	33 (20-48)	32 (21-47)	0.30
	Time from groin puncture to revascularization	63 (42-95)	70 (45-109)	56 (39-85)	<0.001
	Time from onset to revascularization	275 (225-327)	293 (242-341)	264 (210-311)	<0.001
Treatment					
	EVT	299 (50.6)	120 (47.2)	179 (53.1)	0.16
	IVT+EVT	292 (49.4)	134 (52.8)	158 (46.9)	
Anesthesia					
	General	198 (33.5)	83 (32.7)	115 (34.2)	0.69
	non-general	393 (66.5)	171 (67.3)	221 (65.8)	
>3 thrombectomy maneuvers performed		76 (11.2)	43 (16.9)	33 (9.8)	0.010
Acute stenting [h]		81 (13.7)	32 (12.6)	49 (14.5)	0.78
Post-procedure eTICI, n (%)[i]					
	0-1	22/582 (3.8)	8 (3.2)	14 (4.2)	<0.001
	2a	70/582 (12.0)	38 (15.1)	32 (9.7)	
	2b	176/582 (30.2)	95 (37.8)	81 (24.5)	
	2c	115/582 (19.8)	39 (15.5)	76 (23.0)	
	3	199/582 (34.2)	71 (28.3)	128 (38.7)	

Hu X, *et al. J NeuroIntervent Surg* 2023; 15:977–983. doi: 10.1136/jnis-2022-019021

a. IQR interquartile range. ICH intracranial hemorrhage. 65 patients were excluded from this study because of protocol violations or because they did not receive EVT.
b. Scores on the National Institutes of Health Stroke Scale (NIHSS) range from 0 to 42, with higher scores indicating more severe neurologic deficits.
c. Scores on the modified Rankin scale of functional recovery range from 0 (no symptoms) to 6 (death). A score of 2 or less indicates functional independence.
d. The cause of stroke was assessed according to the medical history, clinical features, and results on digital subtraction angiography.
e. Collateral flow was graded with baseline CT angiography using a 4-point scale, with 0 representing absent collateral flow (absent filling of the occluded territory), 1 representing poor collateral flow (less than 50% filling of the occluded territory), 2 representing intermediate collateral flow (between 50% and 100% filling of the occluded territory), and 3 representing excellent collateral flow (100% filling of the occluded territory). Data was not available for 13 patients due to images missing, poor quality of the images.
f. The Alberta Stroke Program Early Computed Tomography Score (ASPECTS) is a measure of the extent of early cerebral ischemia. Scores ranges from 0 to 10, with higher scores indicating fewer early ischemic changes. Shown are values as assessed by the core laboratory.
g. The sites of intracranial artery occlusion were assessed by the core laboratory. Computed tomographic angiographic data at baseline were missing for 3 patients.
h. Acute stenting denotes stenting in either internal carotid artery or middle cerebral artery.
i. The extended Thrombolysis in Cerebral Infarction (eTICI) score (range, 0 [no reperfusion] to 3 [complete reperfusion]) is performed with digital subtraction angiography; successful reperfusion denotes an eTICI score of 2b, 2c, or 3 on the final angiogram.

Table S2. Baseline Characteristics of the 591 included Patients by treatment[a].

Variable	Treatment		*P* value
	EVT alone(n=299)	Combination(n=292)	
Age-yr, median (IQR)	69 (61-76)	69 (61-76)	0.68
Male sex-no. (%)	172 (57.53)	160 (54.79)	0.48
NIHSS-median (IQR)[b]	17 (13-21)	17 (14-22)	0.45
History of ischemic stroke-no. (%)	39 (13.00)	42 (14.38)	0.62
History of atrial fibrillation-no. (%)	145 (48.33)	134 (45.89)	0.55
History of diabetes mellitus-no. (%)	50 (16.67)	65 (22.26)	0.086
History of hypertension-no. (%)	174 (58.00)	179 (61.30)	0.41
History of antiplatelet agent(s) -no. (%)	46 (15.4)	49 (16.8)	0.64
History of anticoagulant agent(s) -no. (%)	19 (6.4)	28 (9.6)	0.15
History of statin -no. (%)	19 (6.4)	28 (9.6)	0.15
Pre-stroke modified Rankin scale, score 1 or 2-no. (%)[c]	24 (8.0)	19 (6.5)	0.48
Median glucose level at hospital arrival (IQR) —mmol/L	7.01 (5.8-8.6)	7.0 (5.91-8.95)	0.32
Systolic blood pressure at hospital arrival -mmHg, median (IQR)	144.5 (129.5-163)	144.5 (131-162)	0.99
Cause of stroke -no. (%)[d]			
Cardioembolic	139 (46.33)	129 (44.18)	0.33
Intracranial atherosclerosis	27 (9.00)	19 (6.51)	
Ipsilateral extracranial obstruction	32 (10.67)	26 (8.90)	
Undetermined	102 (34.00)	118(40.41)	
Collateral score-no. (%)[e]			
0-1	234 (78.00)	231 (79.11)	0.74
2-3	66 (22.00)	61 (20.89)	

Clot burden score		4 (2-5)	4 (2-5)	0.83
Leukoaraiosis		105 (35.1)	83 (28.4)	0.81
ASPECTS-median (IQR)[f]		9 (7-10)	9 (7-10)	0.95
Occlusion site[g]				
	ICA	110 (37.0)	105 (36.1)	0.62
	M1	152 (51.2)	158 (54.3)	
	M2	35 (11.8)	28 (9.6)	
Workflow- min, median (IQR)				
	Time from stroke onset to randomization	170 (129-208.5)	177 (124.5-213.5)	0.76
	Time from randomization to groin puncture	31 (19.5-44.5)	35 (22-49)	0.014
	Time from groin puncture to revascularization	65.5 (45-99)	58.5 (38-93)	0.028
	Time from onset to revascularization	242 (203-286)	249 (200-296)	0.37
Anesthesia				
	General	99 (33.00)	100 (34.25)	0.75
	non-general	201 (67.00)	192 (65.75)	
Stenting				
	Yes	47 (15.67)	36 (12.33)	0.24
	No	253 (84.33)	256 (87.67)	
Post-procedure eTICI, n (%)[h]				
	0-1	12/582 (4.1)	10/582 (3.5)	0.59
	2a	41/582 (14.0)	29/582 (10.0)	
	2b	86/582 (29.4)	90/582 (31.1)	
	2c	59/582 (20.1)	56/582 (19.4)	
	3	95/582 (32.4)	104/582 (36.0)	

a. IQR, interquartile range.

b. Scores on the National Institutes of Health Stroke Scale (NIHSS) range from 0 to 42, with higher scores indicating more severe neurologic deficits.

c. Scores on the modified Rankin scale of functional recovery range from 0 (no symptoms) to 6 (death). A score of 2 or less indicates functional independence.

d. The cause of stroke was assessed according to the medical history, clinical features, and results on digital subtraction angiography.

e. Collateral flow was graded with baseline CT angiography using a 4-point scale, with 0 representing absent collateral flow (absent filling of the occluded territory), 1 representing poor collateral flow (less than 50% filling of the occluded territory), 2 representing intermediate collateral flow (between 50% and 100% filling of the occluded territory), and 3 representing excellent collateral flow (100% filling of the occluded territory).

f. The Alberta Stroke Program Early Computed Tomography Score (ASPECTS) is a measure of the extent of early cerebral ischemia. Scores range from 0 to 10, with higher scores indicating fewer early ischemic changes. Shown are values as assessed by the core laboratory.

g. The sites of intracranial artery occlusion were assessed by the core laboratory.

h. The extended Thrombolysis in Cerebral Infarction (eTICI) score (range, 0 [no reperfusion] to 3 [complete reperfusion]) is performed with digital subtraction angiography; successful reperfusion denotes an eTICI score of 2b, 2c, or 3 on the final angiogram.

Table S3. Baseline Characteristics of the 591 included Patients by non-ICH versus aICH and sICH[a].

	Overall	Non-ICH	Asymptomatic ICH	*P*	Symptomatic ICH	*P*
Age-yr, median (IQR)	69 (61, 76)	70 (61, 76)	69 (61, 75)	0.33	72.5 (63.5, 77)	0.34
Male sex-no. (%)	332 (56.18)	185 (54.90)	129 (58.11)	0.45	18 (56.25)	0.88
NIHSS-median (IQR)[b]	17 (13, 22)	16 (13, 21)	18 (14, 23)	<0.001	21 (16.5, 25)	<0.001
History of ischemic stroke-no. (%)	81 (13.71)	46 (13.65)	33 (14.86)	0.69	2 (6.25)	0.36
History of atrial fibrillation-no. (%)	279 (47.21)	158 (46.88)	105 (47.30)	0.92	16 (50.00)	0.74
History of diabetes mellitus-no. (%)	115 (19.46)	57 (16.91)	49 (22.07)	0.13	9 (28.13)	0.11
History of hypertension-no. (%)	353 (59.73)	201 (59.64)	127 (57.21)	0.57	25 (78.13)	0.04
History of antiplatelet agent (s) -no. (%)	95 (16.07)	54 (16.02)	37 (16.67)	0.84	4 (12.50)	0.60
History of anticoagulant agent (s) -no. (%)	47 (7.95)	21 (6.23)	22 (9.91)	0.11	4 (12.50)	0.33
History of statin administration. -no. (%)	47 (7.95)	21 (6.23)	20 (9.01)	0.22	6 (18.75)	0.02
Pre-stroke modified Rankin scale, score 1 or 2-no. (%)[c]	43 (7.28)	28 (8.31)	13 (5.86)	0.28	2 (6.25)	0.95
Baseline median glucose level at hospital arrival (IQR) —mmol/L	7 (5.9, 8.8)	6.79 (5.80, 8.10)	7.40 (6.20, 9.59)	<0.001	7.60 (6.01, 11.06)	0.04
Systolic blood pressure at admission -mmHg, median (IQR)	145 (130, 163)	146 (130, 163)	144 (130, 161)	0.74	144.5 (134.5, 170)	0.30
Cause of stroke -no. (%)[d]						
Cardioembolic	268 (45.35)	151 (44.81)	100 (45.05)	0.25	17 (53.13)	0.81
Intracranial atherosclerosis	45 (7.61)	32 (9.50)	11 (4.95)		2 (6.25)	
Ipsilateral extracranial obstruction	58 (9.81)	32 (9.50)	23 (10.36)		3 (9.38)	
Undetermined	220 (37.23)	122 (36.20)	88 (39.64)		10 (31.25)	
Collateral score-no. (%)[e]						
0-1	454(78.6)	241(73.03)	186(86.11)	<0.001	27(84.38)	0.16

2-3	124(21.5)	89(26.97)	30(13.89)		5(15.63)	
Leukoaraiosis	188 (31.81)	110 (32.64)	68 (30.63)	0.62	10 (31.25)	0.87
Clot burden score	4 (2, 5)	4 (2, 5)	4 (3, 5)	<0.001	4 (1, 5)	0.08
ASPECTS-median (IQR)[f]	9 (7, 10)	9 (7, 10)	8 (6, 9)	<0.001	8 (7, 9)	0.11
Occlusion site[g]						
ICA	215 (36.56)	108 (32.14)	92 (41.82)	0.06	15(46.88)	0.12
M1	310 (52.72)	189 (56.25)	109 (49.55)		12(37.50)	
M2	63 (10.71)	39 (11.61)	19 (8.64)		5(15.63)	
Workflow- min, median (IQR)						
Time from stroke onset to randomization	173 (127, 211)	170 (124, 202)	176 (132, 221)	0.02	171.5 (126.5, 221)	0.41
Time from randomization to groin puncture	33 (21, 47)	32 (21, 46)	33 (21, 48)	0.41	39 (20, 54.5)	0.35
Time from groin puncture to revascularization	63 (42, 95)	57 (39, 88)	71.5 (46, 109)	<0.001	64 (45, 104.5)	0.18
Time from onset to revascularization	275(225,327)	265(210,316)	292.5(243,340)	<0.001	298.5(243.5,352)	0.05
Treatment						
EVT	299 (50.59)	179 (53.12)	108 (48.65)	0.30	12 (37.50)	0.09
IVT+EVT	292 (49.41)	158 (46.88)	114 (51.35)		20 (62.50)	
Anesthesia						
General	198 (33.50)	115 (34.12)	76 (34.23)	0.98	7 (21.88)	0.16
non-general	393 (66.50)	222 (65.88)	146 (65.77)		25 (78.13)	
>3 thrombectomy maneuvers performed	76 (12.86)	33 (9.79)	37 (16.67)	0.02	6 (18.75)	0.20
Acute stenting [h]	83 (14.04)	51 (15.13)	30 (13.51)	0.59	2 (6.25)	0.27
Post-procedure eTICI, n (%)[i]						
≤2a	92 (15.81)	46 (13.90)	40 (18.26)	0.17	6 (18.75)	0.63
≥2b	490 (84.19)	285 (86.10)	179 (81.74)		26 (81.25)	

a. IQR interquartile range. ICH intracranial hemorrhage. aICH and sICH, asymptomatic and symptomatic ICH.
b. Scores on the National Institutes of Health Stroke Scale (NIHSS) range from 0 to 42, with higher scores indicating more severe neurologic deficits.
c. Scores on the modified Rankin scale of functional recovery range from 0 (no symptoms) to 6 (death). A score of 2 or less indicates functional independence.
d. The cause of stroke was assessed according to the medical history, clinical features, and results on digital subtraction angiography.
e. Collateral flow was graded with baseline CT angiography using a 4-point scale, with 0 representing absent collateral flow (absent filling of the occluded territory), 1 representing poor collateral flow (less than 50% filling of the occluded territory), 2 representing intermediate collateral flow (between 50% and 100% filling of the occluded territory), and 3 representing excellent collateral flow (100% filling of the occluded territory). Data was not available for 13 patients due to images missing, poor quality of the images.
f. The Alberta Stroke Program Early Computed Tomography Score (ASPECTS) is a measure of the extent of early cerebral ischemia. Scores ranges from 0 to 10, with higher scores indicating fewer early ischemic changes. Shown are values as assessed by the core laboratory.
g. The sites of intracranial artery occlusion were assessed by the core laboratory. Computed tomographic angiographic data at baseline were missing for 3 patients.
h. Acute stenting denotes stenting in either internal carotid artery or middle cerebral artery.
i. The extended Thrombolysis in Cerebral Infarction (eTICI) score (range, 0 [no reperfusion] to 3 [complete reperfusion]) is performed with digital subtraction angiography; successful reperfusion denotes an eTICI score of 2b, 2c, or 3 on the final angiogram.

Hu X, *et al. J NeuroIntervent Surg* 2023; 15:977–983. doi: 10.1136/jnis-2022-019021

Table S4. Baseline Characteristics of the 591 included Patients by non-ICH versus HI, PH, and other ICH[a].

	Overall	Non-ICH	HI	*P*	PH	*P*	other ICH	*P*
Age-yr, median (IQR)	69 (61, 76)	70 (61, 76)	69 (60, 76)	0.50	69.5 (63, 76)	0.69	67 (60, 73)	0.33
Male sex-no. (%)	332 (56.18)	185 (54.90)	24 (17.27)	0.31	45 (53.57)	0.83	13 (54.17)	0.94
NIHSS-median (IQR)[b]	17 (13, 22)	16 (13, 21)	18 (14, 23)	0.01	19 (15, 24)	<0.001	16 (12, 21.5)	0.81
History of ischemic stroke-no. (%)	81 (13.71)	46 (13.65)	24 (17.27)	0.31	9 (10.71)	0.48	1 (4.17)	0.31
History of atrial fibrillation-no. (%)	279 (47.21)	158 (46.88)	65 (46.76)	0.98	40 (47.62)	0.90	11 (45.83)	0.92
History of diabetes mellitus-no. (%)	115 (19.46)	57 (16.91)	31 (22.30)	0.17	20 (23.81)	0.14	3 (12.50)	0.78
History of hypertension-no. (%)	353 (59.73)	201 (59.64)	83 (59.71)	0.99	51 (60.71)	0.86	14 (58.33)	0.90
History of antiplatelet agent (s) -no. (%)	95 (16.07)	54 (16.02)	26 (18.71)	0.48	12 (14.29)	0.70	2 (8.33)	0.48
History of anticoagulant agent (s) -no. (%)	47 (7.95)	21 (6.23)	14 (10.07)	0.14	8 (9.52)	0.29	3 (12.50)	0.44
History of statin administration. -no. (%)	47 (7.95)	21 (6.23)	14 (10.07)	0.14	10 (11.90)	0.07	2 (8.33)	1.00
Pre-stroke modified Rankin scale, score 1 or 2-no. (%)[c]	43 (7.28)	28 (8.31)	8 (5.76)	0.34	4 (4.76)	0.27	2 (8.33)	1.00
Baseline median glucose level at hospital arrival (IQR) —mmol/L	7 (5.9, 8.8)	6.79 (5.80, 8.10)	7.4 (6.06, 9.72)	0.01	7.7 (6.48, 10.32)	<0.001	7.15 (6.23, 8.49)	0.31
Systolic blood pressure at admission -mmHg, median (IQR)	145 (130, 163)	146 (130, 163)	142 (131, 158)	0.36	150 (132.5, 170)	0.10	141.5 (122, 155)	0.15
Cause of stroke -no. (%)[d]								
Cardioembolic	268 (45.35)	151 (44.81)	64 (46.04)	0.18	38 (45.24)	0.99	10 (41.67)	0.77
Intracranial atherosclerosis	45 (7.61)	32 (9.50)	5 (3.60)		7 (8.33)		1 (4.17)	
Ipsilateral extracranial obstruction	58 (9.81)	32 (9.50)	14 (10.07)		8 (9.52)		3 (12.50)	
Undetermined	220 (37.23)	122 (36.20)	56 (40.29)		31 (36.90)		10 (41.67)	
Collateral score-no. (%)[e]								
0-1	454(78.6)	241(73.03)	120 (86.33)	<0.001	72 (85.71)	0.02	21 (87.50)	0.12

2-3	124(21.5)	89(26.97)	19 (13.67)		12 (14.29)		3 (12.50)	
Leukoaraiosis	188 (31.81)	110 (32.64)	46 (33.09)	0.92	23 (27.38)	0.35	8 (33.33)	0.94
Clot burden score	4 (2, 5)	4 (2, 5)	4 (2, 5)	0.01	4 (1, 4)	<0.0001	4.5 (2.5, 5)	0.97
ASPECTS-median (IQR)[f]	9 (7, 10)	9 (7, 10)	8 (6, 9)	<0.001	8 (6, 9)	<0.001	9 (7, 10)	0.85
Occlusion site[g]								
ICA	215 (36.56)	108 (32.14)	55 (39.86)	0.24	43 (51.81)	<0.001	6 (25.00)	0.38
M1	310 (52.72)	189 (56.25)	71 (51.45)		34 (40.96)		13 (54.17)	
M2	63 (10.71)	39 (11.61)	12 (8.70)		6 (7.23)		5 (20.83)	
Workflow- min, median (IQR)								
Time from stroke onset to randomization	173 (127, 211)	170 (124, 202)	179 (132, 226)	0.01	173.5 (128, 213.5)	0.23	156.5 (132, 222.5)	0.50
Time from randomization to groin puncture	33 (21, 47)	32 (21, 46)	33 (22, 48)	0.32	32 (20, 50)	0.69	41 (19, 52)	0.37
Time from groin puncture to revascularization	63 (42, 95)	57 (39, 88)	71 (48, 109)	<0.001	66 (43.5, 103.5)	0.06	86 (56.5, 123.5)	0.01
Time from onset to revascularization	275(225, 327)	265(210, 316)	256.5 (206, 300)	0.02	243.5 (204, 303)	0.23	261 (203, 305)	0.22
Treatment								
EVT	299 (50.59)	179 (53.12)	72 (51.80)	0.79	33 (39.29)	0.02	13 (54.17)	0.92
IVT+EVT	292 (49.41)	158 (46.88)	67 (48.20)		51 (60.71)		11 (45.83)	
Anesthesia								
General	198 (33.50)	115 (34.12)	45 (32.37)	0.71	32 (38.10)	0.49	6 (25.00)	0.36
non-general	393 (66.50)	222 (65.88)	94 (67.63)		52 (61.90)		18 (75.00)	
>3 thrombectomy maneuvers performed	76 (12.86)	33 (9.79)	29 (20.86)	<0.001	7 (8.33)	0.68	6 (25.00)	0.05
Acute stenting [h]	83 (14.04)	51 (15.13)	18 (12.95)	0.54	10 (11.90)	0.45	4 (16.67)	1.00
Post-procedure eTICI, n (%)[i]								
≤2a	92 (15.81)	46 (13.90)	24 (17.39)	0.33	13 (15.66)	0.68	6 (26.09)	0.20
≥2b	490 (84.19)	285 (86.10)	114 (82.61)		70 (84.34)		17 (73.91)	

a. IQR interquartile range. ICH intracranial hemorrhage. HI, hemorrhagic infarction. PI, parenchymal hematoma. Other ICH, parenchymal hematoma remote from infarcted brain tissue, intraventricular hemorrhage, subarachnoid hemorrhage, and subdural hemorrhage.
b. Scores on the National Institutes of Health Stroke Scale (NIHSS) range from 0 to 42, with higher scores indicating more severe neurologic deficits.
c. Scores on the modified Rankin scale of functional recovery range from 0 (no symptoms) to 6 (death). A score of 2 or less indicates functional independence.
d. The cause of stroke was assessed according to the medical history, clinical features, and results on digital subtraction angiography.
e. Collateral flow was graded with baseline CT angiography using a 4-point scale, with 0 representing absent collateral flow (absent filling of the occluded territory), 1 representing poor collateral flow (less than 50% filling of the occluded territory), 2 representing intermediate collateral flow (between 50% and 100% filling of the occluded territory), and 3 representing excellent collateral flow (100% filling of the occluded territory). Data was not available for 13 patients due to images missing, poor quality of the images.
f. The Alberta Stroke Program Early Computed Tomography Score (ASPECTS) is a measure of the extent of early cerebral ischemia. Scores ranges from 0 to 10, with higher scores indicating fewer early ischemic changes. Shown are values as assessed by the core laboratory.
g. The sites of intracranial artery occlusion were assessed by the core laboratory. Computed tomographic angiographic data at baseline were missing for 3 patients.
h. Acute stenting denotes stenting in either internal carotid artery or middle cerebral artery.
i. The extended Thrombolysis in Cerebral Infarction (eTICI) score (range, 0 [no reperfusion] to 3 [complete reperfusion]) is performed with digital subtraction angiography; successful reperfusion denotes an eTICI score of 2b, 2c, or 3 on the final angiogram.

Hu X, *et al. J NeuroIntervent Surg* 2023; 15:977–983. doi: 10.1136/jnis-2022-019021

Table S5. Baseline characteristics of the patients of six randomized trials[a].

Characteristics	DIRECT MT		DEVT		SKIP		MR CLEAN NO IV		DIRECT SAVE		SWIFT DIRECT	
Groups	EVT alone (n=299)	Combination (n=292)	EVT alone (n=116)	Combination (n=118)	EVT alone (n=101)	Combination (n=103)	EVT alone (n=273)	Combination (n=266)	EVT alone (n=146)	Combination (n=147)	EVT alone (n=201)	Combination (n=207)
Age-yr, median (IQR)	69 (61-76)	69 (61-76)	70 (60-77)	70 (60-78)	74 (67-80)	76 (67-80)	72 (62-80)	69 (61-77)	70 (61–78)	69 (60–79)	73 (64 to 81)	72 (65 to 81)
Male sex-no. (%)	172 (57.53)	160 (54.79)	66 (56.9)	66 (55.9)	56 (55)	72 (70)	161 (59)	144 (54.1)	78/146 (53%)	88/147 (60%)	96 (48%)	103 (50%)
NIHSS-median (IQR)[b]	17 (13-21)	17 (14-22)	16 (12-20)	16 (13-20)	19 (13-23)	17 (12-22)	16 (10-20)	16 (10-20)	15 (11–20)	15 (10–20)	17 (13-20)	17 (12-20)
Medical history												
Ischemic stroke-no. (%)	39 (13.00)	42 (14.38)	14 (12.1)	19 (16.1)	12 (12)	14 (14)	47 (17.2)	44 (16.5)	26 (18%) with TIA	18 (12%) with TIA	21 (10%)	20 (10%)
Atrial fibrillation-no. (%)	145 (48.33)	134 (45.89)	62 (53.5)	62 (52.5)	57 (56)	64 (62)	86 (31.6)	63 (23.7)	46/146 (32%)	34/147 (23%)	17 (8%)	22 (11%)
Diabetes mellitus-no. (%)	50 (16.67)	65 (22.26)	25 (21.6)	20 (17.0)	16 (16)	17 (17)	40 (14.7)	50 (18.8)	NA	NA	NA	NA
Hypertension-no. (%)	174 (58.00)	179 (61.30)	69 (59.5)	74 (62.7)	61 (60)	61 (59)	121 (44.3)	139/265 (52.5)	86/146 (59%)	89/147 (61%)	121 (60%)	118 (57%)
Smoking-no. (%)	22.3	20.7	28 (24.1)	29 (24.6)	42 (42)	54 (52)	73/263 (27.8)	66/260 (25.4)	NA	NA	NA	NA
Antiplatelet or anticoagulant agent (s) -no. (%)	62 (20.67)	73 (25.00)	NA	NA	35 (35)	35 (34)	109 (39.9)	108 (40.6)	NA	NA	60 (29)	61 (29)
Statin or other lipid lowering agent - no. (%)	19 (6.4)	28 (9.6)	NA	NA	NA	NA	NA	NA	NA	NA	59 (29%)	60 (29%)
Prestroke mRS, score 1 or 2-no. (%)[c]	24 (8.0)	19 (6.5)	6 (5.2) mRS 1	11 (9.3)	17 (17) mRS 1-3	15 (15) mRS 1-3	83/272 (30.5) mRS 1-3	81 (30.4) mRS 1-3	0	0	34 (17%) mRS 1	27 (13%) mRS 1
ASPECTS-median (IQR)[d]	9 (7-10)	9 (7-10)	8 (7-9)	8 (7-9)	7 (6-9)	8 (6-9)	9 (8-10)	9 (8-10)	10 (9–10)	10 (9–10)	8 (7 to 9)	8 (7 to 9)
SBP at hospital arrival-mmHg, median (IQR)	144.5 (129.5-163)	144.5 (131-162)	146 (129-165)	145 (128-168)	158 (132-172)	150 (134-171)	150 (135-167)	150 (130-169)	NA	NA	147 (130-160)	148 (134-165)

Glucose level at hospital arrival-mmol/L, median (IQR)	7.01 (5.8-8.6)	7.0 (5.91-8.95)	6.7 (5.7-8.1)	6.9 (5.9-8.9)	7.5 (2.7) mean (SD)	7.5 (2.9)	6.6 (5.8-7.6)	6.8 (5.9-8.5)	NA	NA	6·5 (5·8-7·5)	6·6 (5·8-7·6)
Cause of stroke-no. (%)[e]												
Cardioembolic	139 (46.33)	129 (44.18)	65 (56.0)	69 (58.5)	67 (66)	72 (70)	NA	NA	NA	NA	70 (35%)	85 (41%)
Intracranial atherosclerosis	27 (9.00)	19 (6.51)	28 (24.1)	23 (19.5)	21 (21)	15 (15)	NA	NA	NA	NA	NA	NA
Ipsilateral extracranial ICA obstruction	32 (10.67)	26 (8.90)	32 (27.6)	32 (27.6)	NA	NA	NA	NA	NA	NA	NA	NA
Undetermined	102 (34.00)	118 (40.41)	15 (12.9)	20 (16.9)	13 (13)	16 (16)	NA	NA	NA	NA	92 (46%)	75 (36%)
Occlusion site[f]												
ICA	110 (37.0)	105 (36.1)	18 (15.5)	17 (14.4)	41 (41)	36 (35)	68 (25)	50(18.8)	33/145 (23%)	31/145 (21%)	57 (28%)	60 (29%)
M1	152 (51.2)	158 (54.3)	95 (81.9)	99 (83.9)	19 (19)	18 (17)	156(57.4)	174(65.4)	80/145 (55%)	83/145 (57%)	133 (66%)	136 (66%)
M2	35 (11.8)	28 (9.6)	3 (2.6)	2 (1.7)	41 (41)	49 (48)	45(16.5)	40(15)	21/145 (14%)	23/145 (16%)	11 (5%)	11 (5%)
Basilar artery	0	0	0	0	0	0	0	0	11/145 (8%)	8/145 (6%)	0	0
Time from stroke onset to randomization-min, median (IQR)	170 (129-208.5)	177 (124.5-213.5)	170 (129-204)	168 (144-216)	129 mean	136 mean	94 (60-137)	93 (71-152)	136 (110-186)	151 (108-204)	123 (99-163)	135 (106-171)
Collateral score-no. (%)[g]												
0–1	234 (78.00)	231 (79.11)	NA	NA	NA	NA	93/267 (34.8)	91/259 (35.1)	NA	NA	NA	NA
2–3	66 (22.00)	61 (20.89)	NA	NA	NA	NA	174/267 (65.2)	168/259 (64.9)	NA	NA	NA	NA
Analysis type[h]	ITT and PP		ITT		PP		ITT		ITT and PP		ITT and PP	

a. IQR, interquartile range. EVT, endovascular treatment. Combination, combined intravenous alteplase and EVT. NA, not applicable.

b. Scores on the National Institutes of Health Stroke Scale (NIHSS) range from 0 to 42, with higher scores indicating more severe neurologic deficits.

c. Scores on the modified Rankin scale of functional recovery range from 0 (no symptoms) to 6 (death). A score of 2 or less indicates functional independence.

d. The Alberta Stroke Program Early Computed Tomography Score (ASPECTS) is a measure of the extent of early cerebral ischemia. Scores ranges from 0 to 10, with higher scores indicating fewer early ischemic changes. Shown are values as assessed by the core laboratory.

e. The cause of stroke was assessed according to the medical history, clinical features, and results on digital subtraction angiography.

f. The sites of intracranial artery occlusion were assessed by the core laboratory.

g. Collateral flow was graded with baseline CT angiography using a 4-point scale, with 0 representing absent collateral flow (absent filling of the occluded territory), 1 representing poor collateral flow (less than 50% filling of the occluded territory), 2 representing intermediate collateral flow (between 50% and 100% filling of the occluded territory), and 3 representing excellent collateral flow (100% filling of the occluded territory).

h. ITT: Intention to treat analyses. PP: Per-protocol analyses. This second analysis of DIRECT MT is a PP one.

	Patient No.	IVT+EVT	EVT	OR（95% CI)	P for interaction
**Age(year)**					
18-60	80	4/42(9.5%)	2/38(5.3%)	1.47(0.22,9.96)	0.54
60-80	231	13/109(11.9%)	8/122(6.6%)	2.23(0.82,6.07)	
≥80	58	3/27(11.1%)	2/31(6.5%)	1.38(0.18,10.73)	
**Sex**					
Male	203	9/97(9.3%)	9/106(8.5%)	0.92(0.33,2.53)	0.04
Female	166	11/81(13.6%)	3/85(3.5%)	5.76(1.42.23.43)	
**History of ischemic stroke**					
No	321	18/152(11.8%)	12/169(7.1%)	1.88(0.84,4.21)	0.95
Yes	48	2/26(7.7%)	0/22(0.0%)	3.84(0.21,71.71)	
**History of diabetes mellitus**					
No	303	13/146(8.9%)	10/157(6.4%)	1.35(0.54,3.41)	0.30
Yes	66	7/32(21.9%)	2/34(5.9%)	4.13(0.75,22.78)	
**History of hypertension**					
No	143	6/74(8.1%)	1/69(1.5%)	5.55(0.64,47.88)	0.26
Yes	226	14/104(13.5%)	11/122(9.0%)	1.46(0.60,3.55)	
**History of antiplatelet**					
No	311	18/154(11.7%)	10/157(6.4%)	1.85(0.79,4.36)	0.90
Yes	58	2/24(8.3%)	2/34(5.9%)	1.93(0.19,19.60)	
**History of anticoagulant agent(s)**					
No	345	16/165(9.7%)	12/180(6.7%)	1.54(0.68,3.48)	0.95
Yes	25	4/13(30.8%)	0/12(0.0%)	10.08(0.41,246.395)	
**History of statin administration**					
No	344	16/165(9.7%)	12/179(6.7%)	1.62(0.70,3.73)	0.21
Yes	27	5/14(35.7%)	1/13(7.7%)	6.55(0.45,95.16)	
**Pre-stroke mRS 1 or 2-no. (%)c**					
No	339	19/164(11.6%)	11/175(6.3%)	2.03(0.90,4.56)	0.57
Yes	30	1/14(7.1%)	1/16(6.3%)	0.80(0.01.43.21)	
**NIHSS**					
2-15	203	9/98(9.2%)	2/105(1.9%)	5.01(1.02.24.60)	0.28
16-19	40	1/16(6.3%)	2/24(8.3%)	0.65(0.04,11.21)	
≥20	126	10/64(15.6%)	8/62(12.9%)	1.37(0.48,3.93)	
**Glucose level at hospital arrival(mmol/L)**					
<7.8	253	10/122(8.2%)	8/131(6.1%)	1.35(0.49,3.69)	0.28
≥7.8	114	10/55(18.2%)	4/59(6.8%)	3.45(0.94,12.68)	
**Systolic blood pressure at admission(mmhg)**					
<140	221	10/100(10.0%)	9/121(7.4%)	1.24(0.47,3.26)	0.06
≥140	148	10/78(12.8%)	3/70(4.3%)	5.23(1.14,24.01)	
**Cause of stroke**					
Cardioembolic	168	12/75(16.0%)	5/93(5.4%)	4.00(1.22,13.14)	0.66
Intracranial atherosclerosis	34	1/12(8.3%)	1/22(4.6%)	2.50(0.12,52.04)	
Ipsilateral extracranial obstruction	35	1/16(6.3%)	2/19(10.5%)	0.53(0.04,7.22)	
Undetermined	132	6/75(8.0%)	4/57(7.0%)	0.95(0.23,3.92)	
**Collateral score**					
2-3	94	5/46(10.9%)	0/48(0.0%)	11.05(0.86,141.76)	0.92
0-1	268	15/130(11.5%)	12/138(8.7%)	1.33(0.58,3.08)	
**Leukoaraiosis**					
No	249	14/127(11.0%)	8/122(6.6%)	1.89(0.73,4.91)	0.96
Yes	120	6/51(11.8%)	4/69(5.8%)	0.26(0.01,6.04)	
**ASPECT score**					
6-9	190	13/90(14.4%)	8/100(8.0%)	2.41(0.87,6.68)	0.96
0-5	42	3/21(14.3%)	1/21(4.8%)	3.12(0.25,38.60)	
**Occlusion site**					
ICA	123	8/58(13.8%)	7/65(10.8%)	1.51(0.47,4.79)	0.92
M1	201	9/103(8.7%)	3/98(3.1%)	2.89(0.73,11.45)	
M2	44	3/16(18.8%)	2/28(7.1%)	0.61(0.05,7.70)	
**Time from onset to radonmisation(min)**					
<173	191	9/90(10.0%)	7/101(6.9%)	1.76(0.55,5.65)	0.54
≥173	178	11/88(12.5%)	5/90(5.6%)	2.34(0.76,7.16)	
**Time from onset to revascularization(min)**					
<275	255	12/121(9.9%)	8/134(6.0%)	1.83(0.67,4.97)	0.80
≥275	114	8/57(14.0%)	4/57(7.0%)	2.17(0.59,8.05)	
**Anesthesia**					
General	122	3/59(5.1%)	4/63(6.4%)	0.05(0.00,1.84)	0.052
Non-general	247	17/119(14.3%)	8/128(6.3%)	3.01(1.20,7.54)	
**Thrombectomy maneuvers>3**					
No	330	15/162(9.3%)	11/168(6.6%)	1.43(0.62,3.30)	0.24
Yes	39	5/16(31.3%)	1/23(4.4%)	8.87(0.94,83.91)	
**Acute Stenting**					
No	316	19/157(12.1%)	11/159(6.9%)	1.87(0.83,4.22)	0.92
Yes	53	1/21(4.8%)	1/32(3.1%)	1.32(0.07,26.01)	
**Post-procedure eTICI**					
≤2a	52	3/20(15.0%)	3/32(9.3%)	1.76(0.30,10.36)	0.31
≥2b	311	17/156(10.9%)	9/155(5.8%)	2.05(0.84,4.96)	

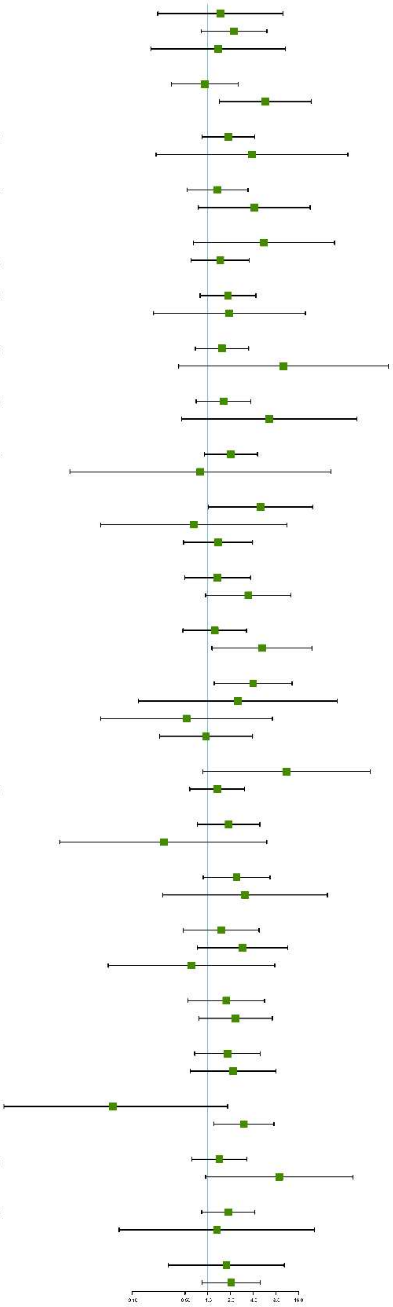

Figure S1. **Forest plot of symptomatic intracranial hemorrhage by subgroups, on as-treated population.**

EVT, endovascular treatment; IVT, intravenous thrombolysis; sICH, symptomatic intracranial hemorrhage.

NIHSS, the National Institutes of Health Stroke Scale (NIHSS), which ranges from 0 to 42 with higher scores indicating more severe neurologic deficits.

ASPECT, Alberta Stroke Program Early Computed Tomography Score, which is a measure of the extent of early cerebral ischemia. Scores ranges from 0 to 10, with higher scores indicating fewer early ischemic changes.

Acute stenting indicated intra-cranial or extra-cranial stenting in Direct-MT.

eTICI, extended Thrombolysis in Cerebral Infarction score, which ranges from 0(no reperfusion) to 3 (complete reperfusion).

Analyses were adjusted by significant predictor factors for sICH in **Table S3.**

Hu X, *et al. J NeuroIntervent Surg* 2023; 15:977–983. doi: 10.1136/jnis-2022-019021

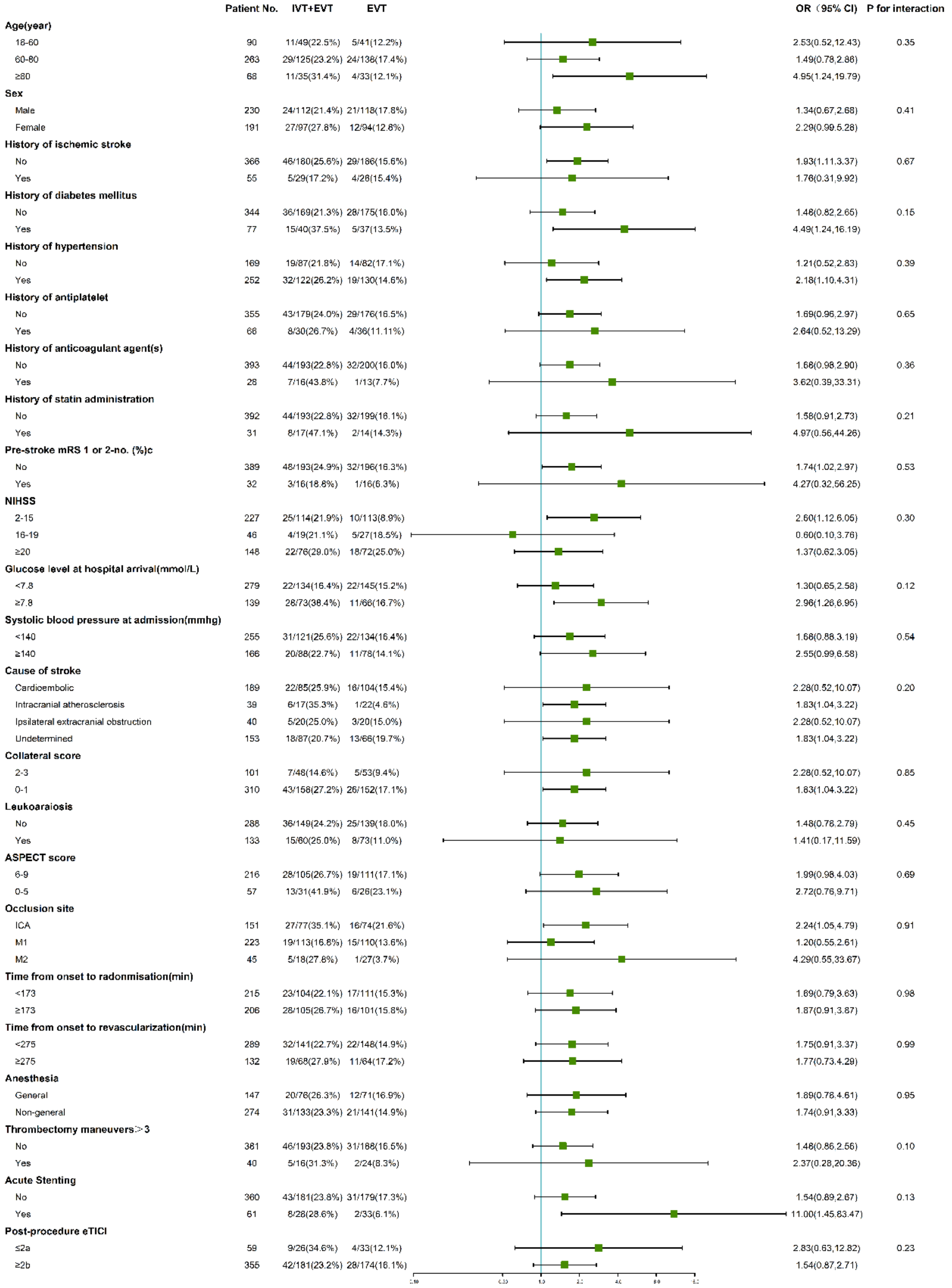

Figure S2. Forest plot of parenchymal hematoma by subgroups, on as-treated population.

EVT, endovascular treatment; IVT, intravenous thrombolysis; PH, parenchymal hematoma

NIHSS, the National Institutes of Health Stroke Scale (NIHSS), which ranges from 0 to 42 with higher scores indicating more severe neurologic deficits.

ASPECT, Alberta Stroke Program Early Computed Tomography Score, which is a measure of the extent of early cerebral ischemia. Scores ranges from 0 to 10, with higher scores indicating fewer early ischemic changes.

Acute stenting indicated intra-cranial or extra-cranial stenting in Direct-MT.

eTICI, extended Thrombolysis in Cerebral Infarction score, which ranges from 0(no reperfusion) to 3 (complete reperfusion).

Analyses were adjusted by significant predictor factors for PH in **Table S4**.
